# Perylene Diimide-Based Fluorescent and Colorimetric Sensors for Environmental Detection

**DOI:** 10.3390/s20030917

**Published:** 2020-02-09

**Authors:** Shuai Chen, Zexu Xue, Nan Gao, Xiaomei Yang, Ling Zang

**Affiliations:** 1 Flexible Electronics Innovation Institute and School of Pharmacy, Jiangxi Science & Technology Normal University, Nanchang 330013, Jiangxi, China; shuai.chen@utah.edu (S.C.); xzx19940717@163.com (Z.X.); gaonan2019@163.com (N.G.); 2 Nano Institute of Utah, University of Utah, Salt Lake City, UT 84112, USA; jaimee@eng.utah.edu; 3 Department of Materials Science and Engineering, University of Utah, Salt Lake City, UT 84112, USA

**Keywords:** environmental detection, perylene diimide, chemosensor, fluorescence, colorimetry

## Abstract

Perylene tetracarboxylic diimide (PDI) and its derivatives exhibit excellent thermal, chemical and optical stability, strong electron affinity, strong visible-light absorption and unique fluorescence on/off features. The combination of these features makes PDIs ideal molecular frameworks for development in a broad range of sensors for detecting environmental pollutants such as heavy metal ions (e.g., Cu^2+^, Cd^2+^, Hg^2+^, Pd^2+^, etc.), inorganic anions (e.g., F^−^, ClO_4_^−^, PO_4_^−^, etc.), as well as poisonous organic compounds such as nitriles, amines, nitroaromatics, benzene homologues, etc. In this review, we provide a comprehensive overview of the recent advance in research and development of PDI-based fluorescent sensors, as well as related colorimetric and multi-mode sensor systems, for environmental detection in aqueous, organic or mixed solutions. The molecular design of PDIs and structural optimization of the sensor system (regarding both sensitivity and selectivity) in response to varying analytes are discussed in detail. At the end, a perspective summary is provided covering both the key challenges and potential solutions for the future development of PDI-based optical sensors.

## 1. Introduction

Developing chemical sensor techniques for trace-level detection of environment hazardous substances, especially heavy metal ions and organic pollutants, remains essential and has drawn increasing research efforts in past decades. Compared with the conventional bench-top analytical instrumental techniques such as chromatography, mass spectrometry, infrared spectrometry, electrochemistry (which normally require expensive, bulky in size and complicated in operation equioment), chemosensors have attracted much more research attention from the chemistry and material science community, mainly due to the unique features of chemosensors, e.g., low-cost, small-size for portability, high sensitivity and selectivity, and quick response for real-time on-site detection [1,2,3,4,5,6]. Among all kinds of chemosensors, optical sensors based on fluorescence or colorimetric signal modulation exhibit superiority regarding system simplicity for facile operation, and non-destructive detection with high sensitivity and selectivity (in some cases, detection can even be visualized by naked-eyes) [4,5,6]. The performances of optical chemosensors rely on rational design of molecular fluorophore or chromophore structures. The main challenge of this molecular design lies in the aspects such as stability against photobleaching, photoluminescence quantum yield, and visible-light absorption, as well as the structural flexibility for substation with side groups that can bind analytes with sufficient affinity and specificity. In most cases, the specific binding is driven by relatively weak host-guest interactions, hydrogen bonding, metal coordination, van der Waals force, electrostatic force, or other noncovalent interactions. Such binding interactions are more or less dynamic, ensuring reversibility of sensing response, a critical parameter for assessing sensor performance.

In the past two decades, perylene tetracarboxylic diimide (PTCDI or PDI) and its derivatives (PDIs), which form a class of high-grade dyes and stable n-type (electron acceptor) semiconductor materials, have drawn extensive research attention for development as fluorescence or colorimetric chemical sensors. PDI molecules possess highly tailorable structures, versatile electronic and optical properties such as excellent electron affinity, strong optical absorption, remarkable monomeric fluorescence with near 100% quantum yields (*ϕ*) in solvents, desirable excited state lifetime, etc. [3,4,5,6]. In particular, PDIs have unique thermal and photochemical stability, i.e. resistance to photobleaching and heat, which both are critical for practical use as optical sensors [4,5,6]. One unique feature of PDI is that the two imide-positions are node in π-orbital, and thus substitution at these positions does not change significantly the electronic property of PDI (e.g., UV-vis adsorption). This provides wide options to change the side groups in order to optimize the binding with analytes (regarding both sensitivity and selectivity), but without changing the absorption wavelength and coefficient, thereby allowing for quantitative comparison of the fluorescence change due to binding to analytes—that’s how a fluorescence sensor works [3]. Additionally, PDI molecules (owing to their rigid planar geometry) are prone to forming supramolecular *H*-aggregates via π-π stacking along with the decreased molecular fluorescence and hypsochromic shift of the absorption band as a result of the strong π-π electronic interaction [3]. Benefiting from such supramolecular assembly–disassembly behavior accompanied with significant optical change (especially fluorescence turn-off/ turn-on and colorimetric change), PDIs are especially suitable for fluorescence and colorimetric sensors with high selectivity and sensitivity [5,6]. 

PDI-based optical sensors can work either as solid-phase thin films or in liquid-phase media. For the former, the sensory materials in solid state must possess considerably high emission intensity with high quantum yield. Through appropriate molecular design and self-assembly control (which in turn depends on the side-group modification), PDIs can be arranged in a way to afford high fluorescence intensity suited for sensor application. PDI thin films (in a format of intertwined nanofibers) were reported from our group as efficient fluorescence sensors for vapor detection of various chemicals, e.g., organic amines, nitroaromatic explosives and phenols, mainly via photoinduced electron transfer (PET) mechanisms [1,2,3]. The porous and open network morphology of PDI films as deposited on a substrate such as glass and silica gel plate allow for expedient diffusion of gas analytes throughout the whole sensor material, and the large surface area intrinsic to the porous nanofiber film further enhances the surface absorption of analytes that plays prominent role in approaching high sensitivity and rapid response for sensors. With the solid phase PDIs mostly employed in vapor sensing, PDI based molecular sensors have mainly been used in solution phase detection of chemicals, particularly environmental hazards and biological species [7,8,9,10,11,12,13,14,15,16,17,18,19,20,21,22,23,24,25,26,27,28,29,30,31,32,33,34,35,36,37,38,39,40,41,42,43,44,45,46,47,48,49,50,51,52,53,54,55,56,57,58,59,60,61]. These molecular sensors can often be fabricated without complex device design. High solubility of PDIs can be feasibly achieved by modifying backbone of PDI with hydrophilic or hydrophobic substituents [5,6]. In addition to the typical PET fluorescence sensing mechanism (via intermolecular or intramolecular process), PDIs based sensors (upon appropriate structure design) can be extended to other sensing modes so as to detect broader range of analytes. Such sensor modes include analyte induced aggregation/disaggregation switch, protonation or chemical reaction induced fluorescence change in liquid-phase. 

So far, several reviews have been published by our groups and others focusing on the solid phase PDI-based sensors including self-assembled nanofibers as chemiresistive sensors for chemical vapor detection (Zang [1,3]), and thin-film fluorescence sensors for detection of volatile organic amines (He [4]). PDI-based supramolecular fluorescent sensors for solution detection of ions have also been nicely reviewed (Jiang [5], Ji [6]). Nevertheless, there is still a lack of good review of PDI-based optical sensors with more specific application in environment detection that covers broad range of pollutants and poisonous chemicals (beyond ions).

**Figure 1:**
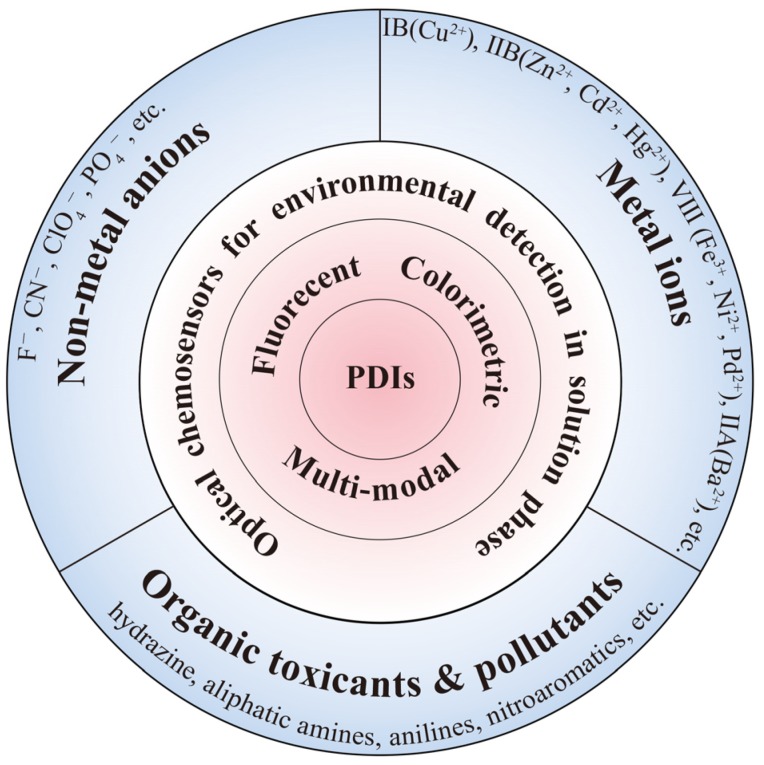
Schematic illustration of main applications of PDI-based optical chemosensors in liquid environmental detection.

In this review, we provide a comprehensive overview of the recent progress in research and development of PDI-based fluorescent and colorimetric sensors, which have proven successful for environment detection covering metal or non-metal ions, amines and other organic pollutants in solution media (Figure 1). With regard to the fact that most PDI-based colorimetric sensors reported so far also function as fluorescent sensors (i.e., binding of analytes causes both absorption and fluorescence change of PDIs), only the sensor systems that rely purely on colorimetric response sensors will be included and discussed in this review as defined as colorimetric sensor. Very often, the PDI-based sensors herein reviewed are not only suited for environment detection, but extensible to chemical monitoring in many other fields such as public security, food safety, biomedicine, healthcare and so on. To this regard, special attention will be given to help the general understanding of molecular design rules in correlation to the corresponding sensing mechanisms. Finally, a conclusion and outlook will be presented with the aim to provide some guidance for the development of new generation of sensors based on the structure-modulable PDIs.

## 2. PDI-Based Fluorescent Sensors for Environment Detection

PDI-based fluorescent sensors can respond to various external stimuli including toxic inorganic ions or organic pollutants in environment, thus realizing sensitive and selective sensing as measured from the change of fluorescence intensity. Sensors for transition metal ions from IB group (Cu^2+^), IIB group (Zn^2+^, Cd^2+^, Hg^2+^) and VIIIB group (Fe^3+^, Ni^2+^, Pd^2+^), and alkaline earth metals IIA group (Ba^2+^), and boron family IIIA group (Al^3+^) have been studied in both aqueous and binary water-organic solutions [7,8,9,10,11,12,13,14,15,16,17,18,19,20,21,22,23,24,25,26,27,28,29,30,31]. Besides metal ions, sensors for other cations like proton (pH detection) have also been developed, particularly for the bio-relevant systems [32,33,34,35,36,37,38]. Similarly, sensors have also been studied for the non-metal anions such as F^−^, CN^−^, ClO_4_^−^ and PO_4_^−^ ions, etc. [9,39,40,41,42,43,44,45]. Sensors for organic pollutants [46,47,48,49,50,51,52,53,54], especially anilines [51,52] and phenols [49,53,54], have been developed in both organic and aqueous solutions, as well as the mixtures. The fluorescent sensing mechanism is often based on analyte-binding-induced assembly-disassembly of PDIs, taking advantage of the sensitive dependence of fluorescence of PDIs on the π-π stacking interaction [5,6,10,18,19,20,21,49,52,53,54]. Many other PDI sensors work through fluorescence turn-on or turn-off mechanism, which relies on an intramolecular PET process [7,8,9,13,15,16,17,22,23,24,25,26,27,28,29,42,43,44,45], or intermolecular electron/proton transfer [51,58,61]. For the turn-on sensor, the original PDI sensor molecule is non-fluorescent or weakly fluorescent because of the efficient fluorescence quenching caused by the electron transfer from the side-binding group (e.g., amine) to the photoexcited state of PDI core. Upon binding or coordination to an analyte (e.g., metal ion, proton, organic compound, etc.), the energy level of the side-group is lowered, thus turning off the intramolecular PET process and turning on the fluorescence of PDI. Such strong fluorescence turn-on can be used a signal modulation to develop sensors for various analytes as described below. Most of the fluorescence sensors are reversable as the analyte binding is non-covalent. As a main parameter in assessing the sensing sensitivity, lowest detection limit (*LDL*) of sensors will be described and discussed in the cases of study mentioned below. It should be noted that all the *LDL* data cited are correlated to the specific testing conditions, especially the concentration of PDIs, and thus not intended to be used for comparison among the different sensor systems.

### 2.1. Metal Ion Sensing

Detection of toxic metal ions is particularly important for monitoring water pollution, food safety and disease diagnosis. In view of the strong tendency of aggregation PDIs molecules through stacking interactions between the π-conjugated skeletons in aqueous media, most studies of PDI-based chemosensors are in organic-aqueous composite systems to maintain the molecular dispersion of PDI sensors [7,8,15,16,17,18,19,20,22,23,24,25,26,27,29,30,31]. There still remain challenges to study the sensing performances in pure water solutions [10,21,32,33,34,35,36,37,38,39]. Most importantly, the fluorescence variations of PDIs are pH-dependent and thus the corresponding sensors should be operated under well-controlled pH of the working solution, for which addition of buffer reagent like (2-[4-(2-hydroxyethyl)-1-piperazinyl]-ethanesulfonic acid) (HEPES) is usually necessary [15,16,20]. The interference from other coexisting metal ions, especially those from the same group in periodic table, should also be considered seriously from the beginning of molecular design of sensors. Both fluorescence turn-on and turn-off mechanisms have been applied in sensing of metal ions [7,8,9,10,11,12,13,14,15,16,17,18,19,20,21,22,23,24,25,26,27,28,29], and a successful sensing highly depends on the molecular structure of PDIs, types of analytes, and solution conditions (e.g., composition, concentration, pH, etc.). 

#### 2.1.1. IB Group (Cu^2+^)

**Figure 2:**
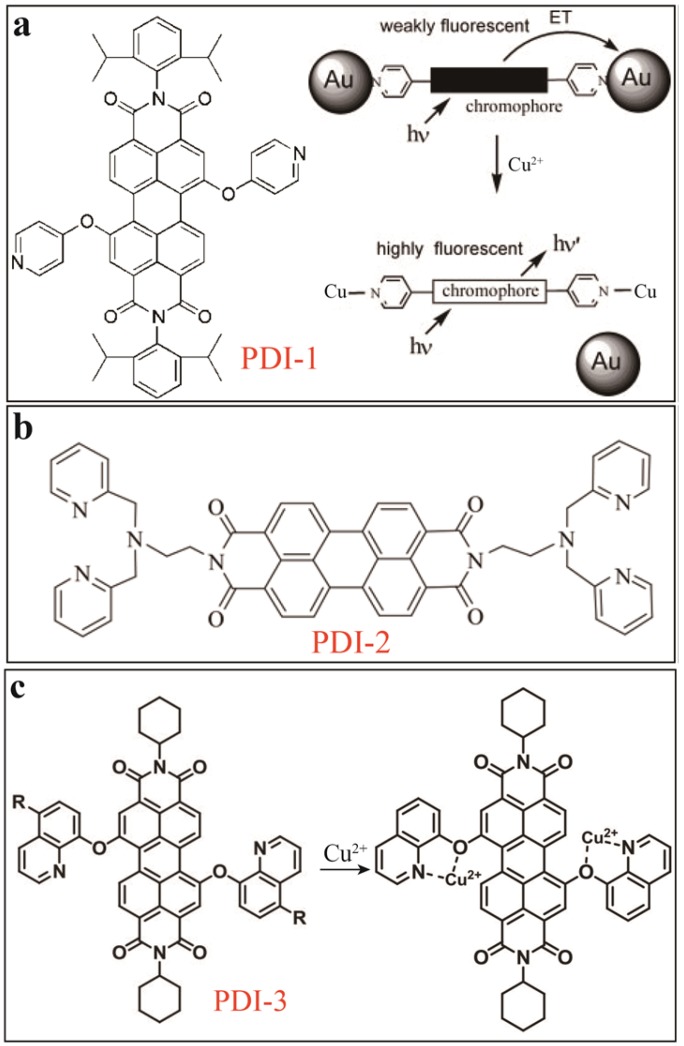
(**a**) Molecular structures of PDI-1, and schematic illustration of sensing mechanism of DPPCA-AuNPs towards Cu^2+^. Reproduced with permission from [7], copyright 2005 WILEY-VCH. (**b**) Molecular structure of PDI-2. (**c**) Molecular structures of PDI-3 and PDI-3-Cu^2+^.

Copper ion causes widely distributed environmental contamination, leading to biological toxicity. PDI-based chemosensors for Cu^2+^ often work as dual-mode involving both colorimetric and fluorescent modulation. The binding groups (as metal ion receptor) are usually amines, and attached at the imide -positions or bay-area of PDIs. Zhu [7] reported a Au nanoparticle (AuNP)-mediated PDI-based fluorescent sensor for detecting Cu^2+^ ion in organic media. At first, the complexation of PDI-1 (Figure 2a) with AuNPs (average diameter ~3 nm) through weak N…Au interactions leads to formation of PDI-1-AuNPs colloids with quenched fluorescence. Upon addition of Cu^2+^, the stronger coordination of Cu^2+^ ion with the pyridyl moiety of PDI-1 results in its fluorescence recovery. A ca. 100-fold increase of PDI-1 emission intensity and also a distinct color change was obtained in a solution of acetonitrile (ACN)/CHCl_3_ (1/9, v/v) with *LDL* of 1.0 µM. Later, a similar dual-mode optical sensor based on PDI-2 (Figure 2b) was reported by Song [8]. With the strong binding affinity of dipicolylethylenediamine (DPEN) moiety with Cu^2+^, sensitive detection of Cu^2+^ was achieved in H_2_O/THF (7/3, v/v) solutions via an effective colorimetric (pink) and fluorometric sensing (near 50% quenching). Furthermore, as reported in 2014 by Singh’s group [9], the recovered or quenched fluorescence of PDI-Cu^2+^ complex can be used as novel sensing platform for next-step on-off sensing process. By attaching 8-hydroxyquinoline at the bay area of PDI as the Cu^2+^ recognition receptor, a sensor based on PDI-3 (Figure 2c) showed significant colorimetric change (coral red to light pink) and fluorescence turn-off upon binding to Cu^2+^. *LDL* of 0.5 µM and 1.0 µM were obtained for the colorimetric and fluorescent sensing, respectively, in CHCl_3_ solutions. The resultant PDI-3-Cu^2+^ complex with 1:2 stoichiometry can be further used as ratiometric sensor to detect CN^−^ ions relying on color change and fluorescence turn-on, for which *LDL* of 10 μM and 8.0 μM were obtained, respectively. Such second-phase of sensing is primarily due to the strong copper-cyanide binding affinity forming stable complex [Cu(CN)_x_], which helps recover the fluorescence of PDI-3.

**Figure 3:**
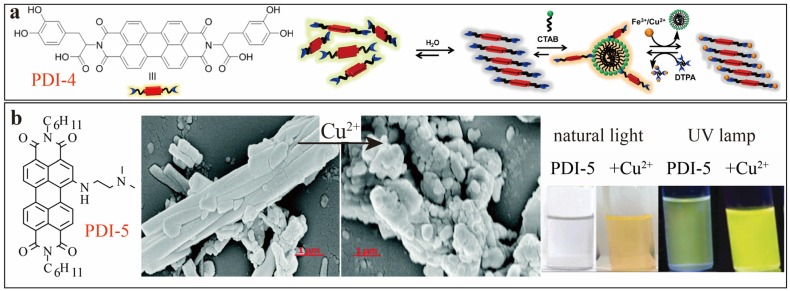
(**a**) Schematic illustration of CTAB and metal ions induced assembly-disassembly process of PDI-4. Reproduced with permission from [10], copyright 2014 American Chemical Society. (**b**) Molecular structures of PDI-5, and the SEM images of their self-assemblies from (10 μM) ACN solution before and after adding Cu(ClO_4_)_2_, as well as photos of color change of 25 μM PDI-5 in ACN under natural light or UV lamp (λ_ex_ = 365 nm) before and after adding 250 μM Cu^2+^. Reproduced with permission from [11], copyright 2016 Royal Society of Chemistry.

Unlike above PET mechanisms, Govindaraju [10] introduced in 2014 a host−guest interaction-driven assembly-disassembly sensor mechanism based on amphiphilic PDI-4 (Figure 3a) substituted with hydrophilic amino acid dihydroxyphenylalanine (L-DOPA) moieties. L-DOPA affords strong binding towards Fe^3+^/Cu^2+^ ions in aqueous solutions, enabling efficient fluorescence sensing with assistant of micellar system formed by cationic surfactant cetyltrimethylammonium bromide (CTAB). The CTAB micelle helps dissolve PDI-4, thus allowing for molecular dispersion that recovers the fluorescence of PDI molecules. When Fe^3+^/Cu^2+^ ions are present, the stronger (more competitive) binding with L-DOPA pulls the PDI-4 molecules out of the micelle, leading to formation of aggregate and fluorescence quenching. With addition of diethylenetriaminepentaacetic acid (DTPA), an even stronger binding ligand to Fe^3+^/Cu^2+^ ions, the aggregate of PDI-4 would be dissociated again, and as a result, the fluorescence of PDI got recovered by being associated back into the micelle of CTAB. Singh [11] later reported a similar sensor approach based on modulation of molecular assembly of PDIs. As shown in Figure 3b, binding with Cu^2+^ triggered morphology disintegration from nanorods to break the spherical aggregate of PDI-5 in ACN solution. A visual color change from colorless to yellow and a strong fluorescence turn-on response (from weakly emissive solution to bright yellow emission under UV-365nm lamp) was observed upon addition of higher equivalents of Cu^2+^ ions (Figure 3b). The strong fluorescence turn-on (approximately 310% increase in the emission intensity in the presence of 2:1 molar ratio of Cu^2+^ to PDI-5) is due to the fact that Cu^2+^ complexation with the bay-substituted group (also a strong electron donor) inhibits the intramolecular PET quenching process, i.e., turning on the fluorescence as discussed above. Uniquely, the PDI-5 based sensor demonstrated multiple sensing modes including ‘on–off–on’, ‘off–on–off’ and ‘off-on’ fluorescence switching, and other mechanisms such as logic gates, and complementary logic circuits in the solution or solid form. Moreover, PDI-5 and PDI-5-Cu^2+^ (2:1) complex showed significant fluorosolvatochromism that can be used to differentiate organic solvents.

#### 2.1.2. ⅡA Group (Ba^2+^) and ⅢA Group (Al^3+^)

**Figure 4:**
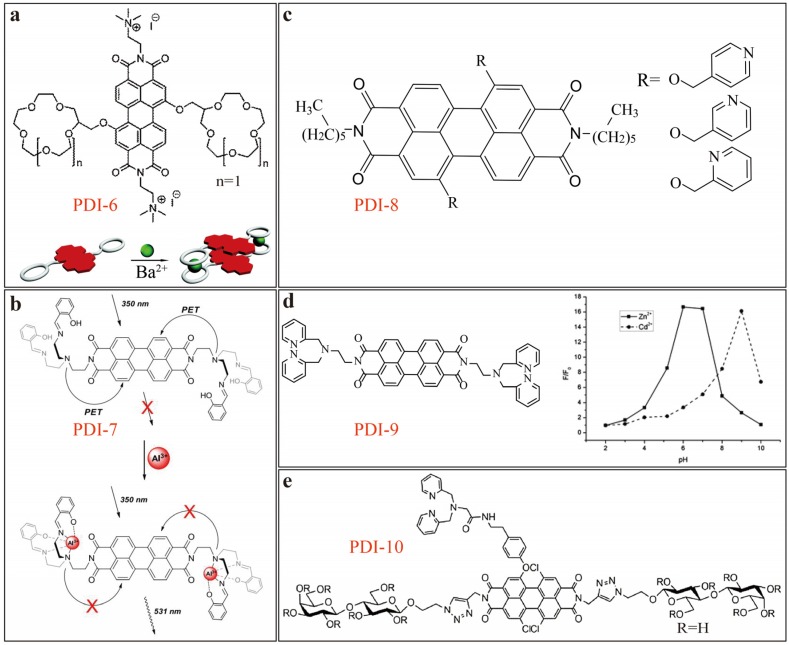
(**a**) Molecular structures of crown ether functionalized PDIs and the dimer formed via complexation between crown ether and Ba^2+^ ion. Reproduced with permission from [12], copyright 2015 Royal Society of Chemistry. (**b**) Fluorescence turn-on sensing mechanism of PDI-7 towards Al^3+^. Reproduced with permission from [13], copyright 2014 Elsevier. (**c**) Molecular structures of three PDIs modified with three pyridine isomers. (**d**) Molecular structure of PDI-9, and the effect of pH on their fluorescence response to Zn^2+^ and Cd^2+^ in ACN/HEPES buffer (1/1, v/v) solutions. Reproduced with permission from [15], copyright 2013 Royal Society of Chemistry. (**e**) Molecular structure of PDI-10.

Würthner [12] reported on a unique fluorescence turn-off sensor based on PDI 6 modified with 15-crown-5 ether (Figure 4a) that demonstrated selective detection of Ba^2+^ ion. The sensing mechanism is due to the metal ion-induced self-assembly of PDI molecules, which in turn causes fluorescence quenching (turn-off). Coordination between Ba^2+^ and 15-crown-5 ether enables formation of H-type aggregates of PDIs, while the side-group modification at the imide-positions help increase the solubility of PDI-6, thus facilitating the sensor processing and performance (molecular solubility is essential to assure the high fluorescence intensity).

Aluminum is the third most prevalent element in the Earth’s crust, and the toxicity of Al^3+^ (especially at accumulated level) causes environmental issues. Relying on intramolecular PET mechanism, Malkondu [13] reported a fluorescence turn-on senor employing PDI-7 (Figure 4b) modified with di(2-(salicylideneamino))ethylamine (DSEA) at the imide-positions as the binding receptor for metal ions. The PDI-7 sensor was found to capable of detecting Al^3+^ ion in ACN solutions with both high sensitivity and selectivity (against other coexisting metal ions). With 1.0 µM of PDI-7 used, *LDL* of 0.33 µM was obtained for Al^3+^ ion in ACN solutions. The strong complexation between DSEA and Al^3+^ leads to increase in fluorescence intensity via inhibiting the intramolecular PET process between DSEA and PDI core. Anthony [14] reported a series of PDIs substituted with pyridine isomers at the bay area, namely PDI-8 (Figure 4c), and these PDIs demonstrated selective colorimetric and fluorescent sensing of Fe^3+^ and Al^3+^ ions from other interference cations in dimethylformamide (DMF). It was found, however, that it was not the above-mentioned metal coordination but the common Lewis acidic character of Fe^3+^ and Al^3+^ ions that was responsible for the observed sensing. The protonation of base pyridine nitrogen atoms on PDI-8 in the presence of the two acidic ions hinders the intramolecular charge transfer band from the nitrogen lone pair electrons to the PDI core, and thus resulting in change in electronic structure and fluorescence quenching.

#### 2.1.3. ⅡB Group (Zn^2+^, Cd^2+^ and Hg^2+^)

Zinc ion is the second most abundant transition metal ions in the body of human, and cadmium ion is typical heavy meatal toxicity in environment. Dipicolylamine (DPA) moiety has been widely used as complexant to Zn^2+^ with a 2:1 binding stoichiometry, but in most cases, the related fluorescent sensors can’t selectively recognize between Zn^2+^ and Cd^2+^ ions, which both belong to the same ⅡB group in the periodic table and share the same chemical coordination property. Shangguan [15] reported a fluorescent sensor based on PDI-9 (Figure 4d) that can selectively detect Zn^2+^ and Cd^2+^ ions, for which the discrimination is realized through adjustment of pH (i.e., fluorescence turn-on is maximal at pH 6.0–7.0 for Zn^2+^, but at pH 9.0 for Cd^2+^). The fluorescence turn-on was rapid in response, and highly sensitive with *LDL* of 32 nM determined for Zn^2+^ and 48 nM for Cd^2+^. The different dependence of fluorescence enhancement on pH observed for Zn^2+^ and Cd^2+^ ions is due to the optimal pH that is required for maximizing the complexation affinity (thermodynamic equilibrium). Strong complexation of DPA with Zn^2+^ or Cd^2+^ ions lowers down the energy level of the long pair of electrons on the amine, thus blocking the intramolecular PET process, and turning on the florescence. Moreover, since the two DPA groups are substituted at the imide-position that is a node in the wavefunction of the -orbital of PDI, protonation or deprotonation of DPA caused by pH change does not change the electronic structure of PDIs, i.e., the absorption spectra remains unchanged. This is highly conducive to the spectral measurement and quantitative comparison for fluorescence sensing, for which the turn-on efficiency is dependent on the fluorescence intensity that in turn is dependent on the absorption at certain excitation wavelength. 

A fluorescence turn-on sensor selective for Cd^2+^ was reported recently by Li [16]. The sensor is based on a relatively complicated PDI structure, namely PDI-10 (Figure 4e). This sensor showed high sensitivity with a *LDL* of 0.52 μM determined in tris(hydroxymethyl)aminoethane hydrochloride (Tris-HCl) buffer solution (Ph = 7.3); similar but slightly lower sensitivity was obtained in HEPES buffer solution. Although PDI-10 also employs DPA as the binding moiety to metal ion, but it is attached it at the bay-area of PDI skeleton. The two imide-positions are substituted with two highly hydrophilic lactose side-chains, enabling high solubility in aqueous solution. Also, the biocompatibility of lactose gives more chance for PDI-10 to be used in biological aqueous solutions or living cells for monitoring the metal ions of interest. And measurement of pH-dependent emission spectra indicates that PDI-10 possesses stable but weak fluorescence intensity under wide range of pH 3.0–9.5. The low fluorescence intensity is mainly due to the same intramolecular PET between DPA and the PDI core as discussed above. Within the optimal pH conditions, high selectivity can be achieved for Cd^2+^, particularly against the analogous ions like Zn^2+^. The unique selectivity towards Cd^2+^ ion is likely due to the additional coordinated complex interactions of the DPA moiety, which in turn is caused some π-π stacking interactions between PDI backbones. 

**Figure 5:**
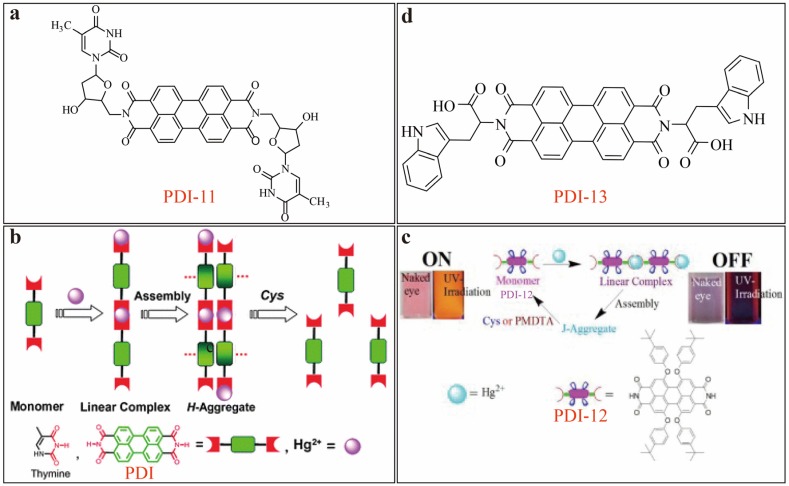
(**a**) Molecular structure of PDI-11. (**b**) Scheme of Hg^2+^–coordination induced aggregation of PDIs and the dissociation caused by competitive complexation of cysteine. Reproduced with permission from [18], copyright 2010 Royal Society of Chemistry. (**c**) Scheme of Hg^2+^-coordination induced *J*-aggregation of PDI-12 and cysteine-induced dissociation. Reproduced with permission from [19], copyright 2014 Elsevier B.V. (**d**) Molecular structure of PDI-13.

Mercury (II) ions have much more serious toxicity than other heavy metal ions and cause widespread contamination in water system and soil. Moreover, Hg^2+^ ion can be accumulated through the food chains. Both fluorescence turn-off and turn-on sensors have been developed based on PDIs, and many of them have demonstrated high efficiency in detection of Hg^2+^. In 2008 [17], our group first reported an ultra-selective Hg^2+^ sensor relying on fluorescence quenching mechanism. The sensor molecule is a PDI substituted with two thymine moieties (PDI-11, shown Figure 5a) that afford selective complexation with Hg^2+^ ion. The 1:2 complexation causes polymerization (aggregation) of PDIs, which in turn leads to significant fluorescence quenching. Under an optimal condition in DMF/H_2_O (7/3, v/v) solutions, a *LDL* of 5 nM was obtained, which allowed for monitoring the mercury pollution even down to the level as low as required for drinking water. The aggregation induced fluorescence quenching is in sharp contrast to the above-mentioned fluorescence quenching mediated by electron transfer process. Addition of acid can break up the thymine–Hg^2+^–thymine coordination by competitive protonation of the thymine moiety (which in turn lowers the complexing capability of thymine). As a result, the fluorescence of PDI-11 can be fully recovered, making the sensor system reversible and recyclable. Later in 2010, another PDI sensor based on similar thymine–Hg^2+^ complexation induced aggregation (Figure 5b) was reported by Jiang’s group [18], though the thymine was linked to the PDI through hydrogen bonding (rather than permanent covalent bond). Interestingly, the non-covalent thymine-PDI structure not only allows for sensitive detection of Hg^2+^ ion, but also cysteine, a thiol–containing amino acid. Upon addition of cysteine, the stronger binding with Hg^2+^ ion will dissociate the complexation of thymine-Hg^2+^, thus breaking up the aggregation of PDIs, and turning on the fluorescence. Such turn-on sensing has proven sensitive for detection of cysteine, with a LDL down to 9.6 nM in DMF/H_2_O (9/1, v/v) solution. In another example, Lin and co-workers [19] developed a dual-mode sensor (involving fluorescent and colorimetric response) for detecting Hg^2+^ and cysteine via *J*-aggregation and deaggregation of PDI-12 (Figure 5c), for which LDL of 36.6 and 91.3 nM were determined in tetrahydrofuran (THF)/H_2_O (2/1, v/v, at pH = 7) solution for Hg^2+^ and cysteine, respectively. Similar fluorescence turn-on sensors have been developed and employed in detecting thiol-contained bio-compounds (e.g., cysteine, homocysteine and glutathione) in dimethylsulfoxide (DMSO)/HEPES buffer (1/19, v/v; pH = 7.4) solutions. One such example as reported by Yilmaz [20] is PDI-13 (Figure 5d), which is modified with tryptophan side-chains. The preformed Hg^2+^-PDI-13 complex (aggregate) is not fluorescent, but can be turned on in fluorescence upon addition of thiol-contained compounds, which in turn dissociate the PDI aggregate through competitive binding with /Hg^2+^ ions as discussed above. 

**Figure 6:**
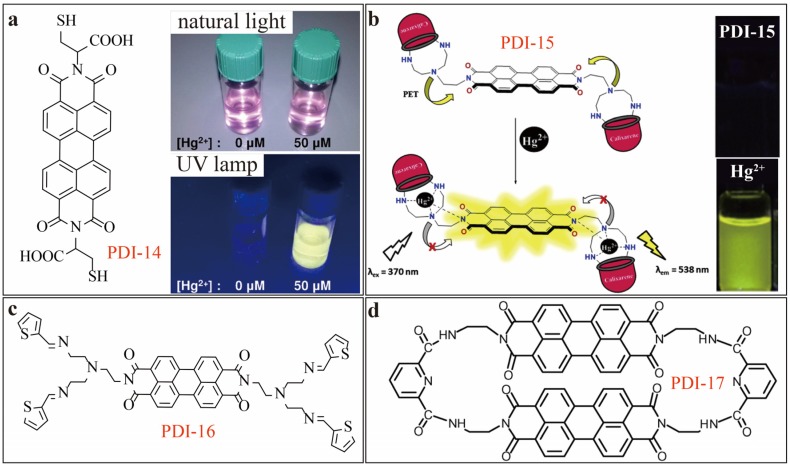
(**a**) Molecular structure and photographs of PDI-14 (30 µM) in water in the absence and presence of Hg^2+^ under ambient light or UV-365 nm light. Reproduced with permission from [21], copyright 2016 Wiley. (**b**) Schematic showing the mechanism of Hg^2+^-binding induced fluorescence enhancement and photographs of the fluorescence of PDI-15 solution before and after addition of Hg^2+^. Reproduced with permission from [22], copyright 2015 Elsevier B.V. (**c**) Molecular structure of PDI-16. (**d**) Molecular structure of PDI-17.

For fluorescence enhancement (turn-on) sensing, Galeotti [21] developed a simple sensor, PDI-14 (Figure 6a), a PDI substituted with two cysteine at the imide-positions. PDI-14, like many other PDIs, tends to aggregate in aqueous solution and thus remains weak or non-fluorescent. Upon complexing with Hg^2+^ (in 1:2 stoichiometric ratio), the molecular aggregate of PDIs can be dissociated, turning on the strong yellow molecular fluorescence as illustrated in Figure 6a. Under an optimal condition, a LDL of 0.1 µM (20 ppb) can be obtained for Hg^2+^. Remarkably, PDI-14 sensor can be operated in pure water system without any assistance of organic solvents like DMF or THF that are often needed for optimal performance of aqueous fluorescence sensors. In 2015, Erdemir [22] reported on PDI-15 sensor (Figure 6b) by introducing two calix[4]arene units into the imide-positions of PDI. Under pH range of 5.5—7.5 in DMF/H_2_O (19/1, v/v) solutions, strong fluorescence turn-on (over 14-fold increase in intensity) was observed when PDI-15 forms a 1:2 complex with Hg^2+^ ion. In contrast to the disassembly induced enhancement of fluorescence as mentioned above, the observed fluorescence enhancement of PDI-15 is otherwise due to inhibition of the intramolecular PET process from calix[4]-aza-crown moieties to the PDI core. This sensor showed both high selectivity and sensitivity, with *LDL* of 0.56 μM determined for Hg^2+^ when 2 µM of PDI-15 used. Erdemir et al. also designed another molecule, PDI-16 (Figure 6c) [23], which bears di(2-thiopheneylimino)ethylamine units as the metal ion coordination receptor. Under the similar solution condition, PDI-16 (used at 20 µM) demonstrated 20-fold enhancement in fluorescence upon binding to Hg^2+^ ions. This gives a *LDL* of 2.20 μM. Another interesting fluorescence sensor PDI-17 (Figure 6d) was reported by Chen [24]. Surprisingly, in phosphate buffer DMSO/H_2_O (1/1, v/v, pH = 7.0) solutions, PDI-17 showed both high sensitivity with much lower *LDL* of 0.01 μM, and selectivity towards Hg^2+^ via dual-wavelength (ratiometric) fluorescence modulation. Basically, upon binding to Hg^2+^, the fluorescence gets increased at 365 nm, while the fluorescence at 557 nm gets decreased. Ratiometric sensing usually gives additional enhancement of detection sensitivity by incorporating both the signal changes. PDI-17 can work in both acidic solution and neutral buffer solution, even in the presence of human serum albumin (HSA). The two connected PDI fluorophore units in semirigid chair-shaped conformation facilitates the complexation between 2,6-bis(aminoethyl)-pyridine and Hg^2+^ ions. The nitrogen atoms of 2,6-bis(aminoethyl)-pyridine function as both ion receptor and electron donor enabling the intramolecular PET process.

#### 2.1.4. ⅧB Group (Fe^3+^, Ni^2+^ and Pd^2+^)

**Figure 7:**
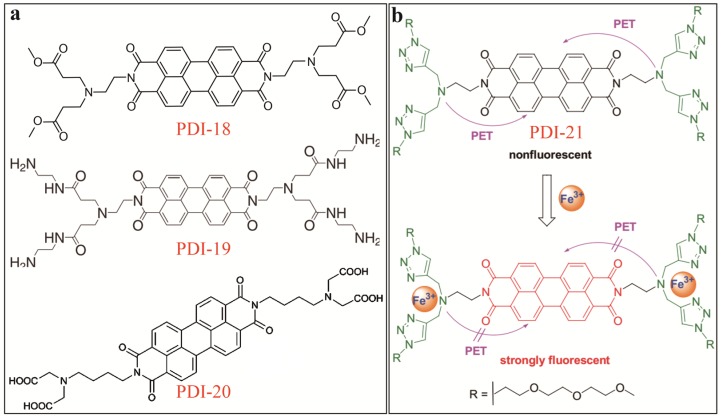
(**a**) Molecular structures of PDI-18 and PDI-19, and PDI-20. (**b**) Schematic illustration of Fe^3+^-binding induced fluorescence turn-on mechanism of PDI-21. Reproduced with permission from [27], copyright 2014 Wiley.

Fe^3+^ is a typical biochemical ion essential for supporting human health. Bojinov [25] reported on two PDI fluorescence turn-on sensors for Fe^3+^ in aqueous solution, PDI-18 and PDI-19 as illustrated in Figure 7a. PDI-18 and PDI-19 are PDIs modified at the imide-position with tetraester- and polyamidoamine moieties, both in dendritic swallow-tail conformation, which are strong binding receptor to metal ions and protons. Upon complexation with metal ions, the electron donating power of the side groups is decreased, thus blocking the intramolecular PET and turning on the fluorescence of PDI core. The two PDI sensors demonstrated efficient fluorescence enhancement towards Fe^3+^ in DMF/H_2_O (1/1, v/v) solutions under a wide pH range from acidic to neutral. The turn-on efficacy of PDI-18 is about 30 times higher than PDI-19 (under certain pH), mainly due to the conformational and scaffold difference in binding ligands of the two sensors. Similar fluorescence enhancement sensing for Fe^3+^ was also reported by Hirsch [26] with a PDI-20 (Figure 7a) as the molecular sensor. PDI-20 contains two ethylenediaminetetraacetic acid (EDTA) side groups for metal ion complexation. However, all the sensors shown in Figure 7a can hardly distinguish between Fe^3+^ and other interference ions like Cu^2+^, Al^3+^ and among others, due to the generic strong binding of the side groups towards metal ions. Significant improvement of the sensing selectivity was made by Liu [27] using bis((1,2,3-triazol-4-yl)methyl)amine (DTA) as the binding ligand for Fe^3+^, which can form stable five-member ring complex with Fe^3+^. Such sensor, namely PDI-21 as shown in Figure 7b, demonstrated much improved detection selectivity towards Fe^3+^ in ACN/H_2_O (1/1, v/v) solutions.

**Figure 8:**
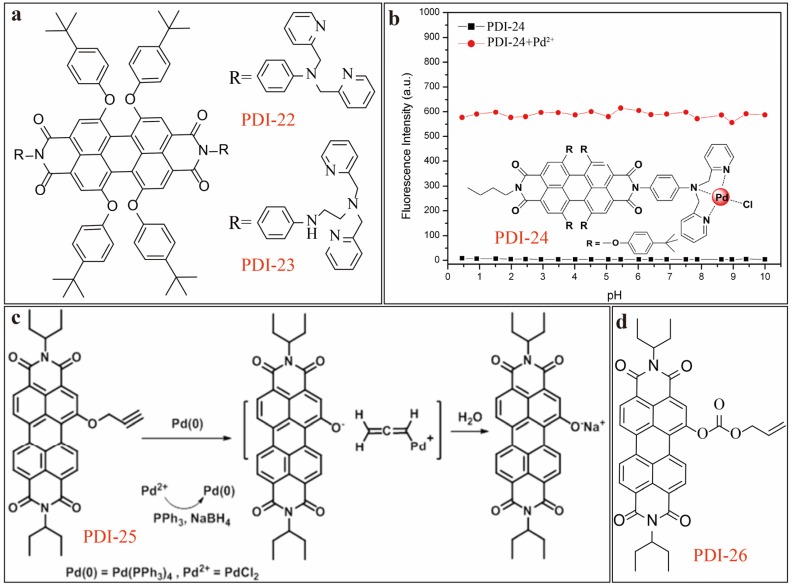
(**a**) Molecular structures of PDI-22 and PDI-23. (**b**) pH-resistant fluorescence intensity of PDI-24 (5 µM) in DMF/H_2_O (7:1, v/v) before and after binding with Pd^2+^ under excitation wavelength of 540 nm. Reproduced with permission from [29], copyright 2014 Elsevier B. V. (**c**) Schematic mechanism of Pd^0^ triggered depropargylation reaction of PDI-25. Reproduced with permission from [30], copyright 2016 Royal Society of Chemistry. (**d**) Molecular structure of PDI-26.

As a common metal ion in environment, Ni^2+^ causes sensing interference to Fe^3+^, especially for the optical chemosensors that are based on chemical binding or complexation. Aiming to improve the discrimination between Fe^3+^ and Ni^2+^, Li [28] developed PDI-22 and PDI-23 (Figure 8a) as fluorescence turn-on sensor for selective detection of the two metal ions. The new sensor molecules are based on PDI backbone but with four substitutions at the bay area. Two binding groups, N-dipicolylamine aniline (DPA) and N-ethyldipicolylamine aniline (EDPA), are attached at the two imide-positions. PDI-22 showed high selectivity toward Ni^2+^, while PDI-23 was highly selective toward Fe^3+^. Both tests were carried out in the presence of Fe^3+^ or Ni^2+^ (acting as interference each other) and various other metal ions including Zn^2+^, Cd^2+^ and Cu^2+^, which are all common interference in environment. The major reason behind the unique selectivity for Fe^3+^ vs. Ni^2+^ is likely due to the different molecular structure of binding groups. With an extra diamino ethylene group introduced between DPA and the phenyl bridge, PDI-23 becomes more preferable for binding with Fe^3+^ than Ni^2+^. The fluorescence enhancement factor of PDI-23 to Fe^3+^ was as high as 138. Later in 2014 [29], the same lab designed an asymmetric PDI-24 (Figure 8b) (with *ϕ* = 0.0041) based on the same PDI backbone with one imide side substituted with DPA as binding receptor and other side with butyl group. PDI-24 demonstrated remarkable fluorescence turn-on sensing for Pd^2+^ ion in DMF/H_2_O (7/1, v/v) solutions with both high sensitivity and selectivity. Fluorescence enhancement factor of 120 and a LDL of 7.32 nM were obtained under optimal testing conditions. More importantly, PDI-24 and its complex with Pd^2+^ exhibited excellent stability regarding the constant fluorescence intensity measured in a wide range of pH (2–10) as shown in Figure 8b. This is particularly essential for detecting Pd^2+^ ions in natural environment, wherein Pd^2+^ ions are usually accumulated through bio-processes. Most of the fluorescence sensors can hardly maintain such strong pH resistance in wide range. Singh [30] reported on an indirect method for detecting Pd^2+^ ions as shown in Figure 8c. The sensor is based on PDI-25, which undergoes self-assembly forming nanosphere and nanorod structures in THF/H_2_O (1/1, v/v) and DMSO /H_2_O (1/9, v/v), respectively. Upon reaction with palladium metal (Pd^0^) via depropargylation, the large aggregates of PDI-25 leads break up into smaller spherical aggregates, which leads to absorption change in near-IR region (around 710 nm) and quenching of fluorescence emission at 630 nm as observed in DMSO/H_2_O solutions. A LDL of 6.6 nM can be estimated for the detection of Pd^0^ from this testing. Since, Pd^2+^ can be easily converted to Pd^0^ by reducing reagent like NaBH_4_, the sensing approach shown in Figure 8c can be feasibly adapted for detecting Pd^2+^ in aqueous media, taking the advantage of high selectivity intrinsic to the depropargylation reaction. Later in 2018 [31], the same lab reported on a further optimized sensor, PDI-26 (Figure 8d), by changing the side-reactive group to allylcarbonate. The new sensor demonstrated the same response to Pd^0^ in ACN/H_2_O (1/1, v/v) and DMSO/H_2_O (7/3, v/v) media, via a Pd^0^-induced deallylation process, which occurs through Tsuji–Trost allylic oxidation followed by subsequent decarboxylation. It was found that the aggregates of PDIs demonstrated faster response to Pd^0^ than the molecularly dissolved PDIs, implying that the Pd^0^-mediated PDI sensor can be used in both solution and solid phase. 

### 2.2. Non-Metal Anion Sensing

As seen above, solution pH plays crucial role in affecting the assembly-disassembly and PET-controlled fluorescence sensing of PDIs. Especially for the detection of non-metal anions, most of the PDI-based fluorescent sensors used are relied on pH dependent (H^+^ variation) PET process [32,33,34,35,36,37,38]. In these cases, water-solubility is a primary requirement for design and synthesis of PDIs sensor molecules. In contrast to the numerous biological probing applications of PDIs-based optical sensors towards pH or biomolecules in living cells etc., the same type of PDI sensors have much less studied in environmental detection of non-metal anions, particularly those from VA group (F, Cl) and VIIIA group (N, P) elements were reported [39,40,41,42,43,44,45]. 

#### 2.2.1. Fluoride Ion (F^−^)

**Figure 9:**
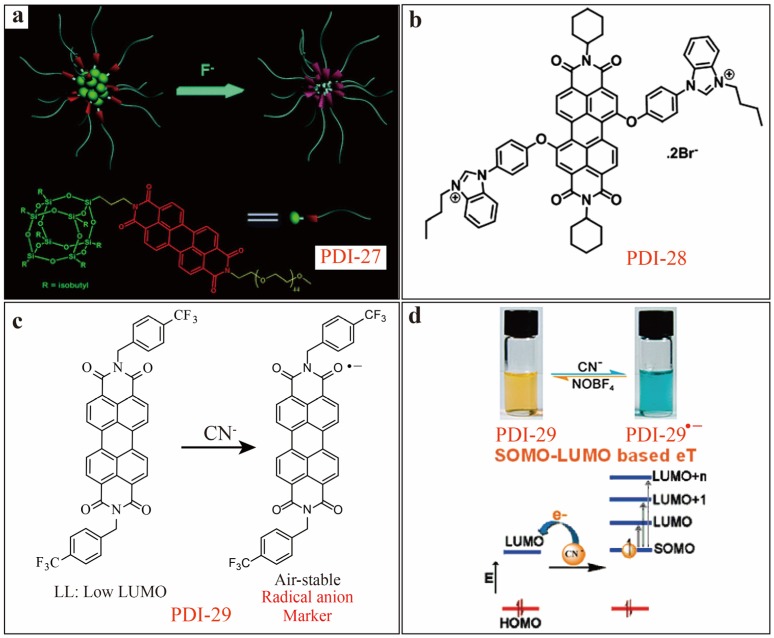
(**a**) Schematic illustration of the F^−^ induced self-assembly of PDI-27 polymeric nanoparticles in water. Reproduced with permission from [39], copyright 2013 Royal Society of Chemistry. (**b**) Molecular structure of PDI-28. (**c**) Schematic representation of CN^−^ sensing by SET-based reaction of PDI-29, and (**d**) its signal transduction pathway and photographs showing colorimetric reversible response of PDI-29 to CN^−^ and oxidizing agent NOBF_4_^−^ in THF. Reproduced with permission from [41], copyright 2010 American Chemical Society.

F^−^ remains crucial for health, deficiency or excess of F^−^ can cause medical problem. It is important to monitor the concentration of F^−^ in environmental and bio-related aqueous system. In 2013, Bai [39] reported on a PDI-based fluorescence quenching sensor, namely PDI-27 (Figure 9a), which is substituted at the imide positron with polyhedral oligomeric silsesquioxane (POSS). Self-assembly of PDI-27 leads to formation of nanoparticles with POSS aggregate in the core. The nanoparticles thus formed demonstrated rapid detection of F^−^ in aqueous solution with both high selectivity and sensitivity. The selective sensing is based on the specific reaction between F^−^ and POSS, a partial or complete hydrolysis of POSS nanocages by F^−^, which in turn induces aggregation of PDIs in the nanoparticle core, thus resulting in efficient fluorescence quenching. A *LDL* as low as 10 µM was obtained under the best testing condition as tried, there is room to further to improve the detection limit through signal optimization. The unique features of PDI-27 (in comparison to other chemosensors) include the rapid sensing response (within 10 s) to F^−^ ions even in pure water, rich binding sites of POSS for F^–^, and facile synthetic accessibility and structure flexibility for self-assembly.

#### 2.2.2. Perchlorate Ion (ClO_4_^−^)

Perchlorates are of wide presence in water and soil, and may impose serious health problem at elevated concentration, as ClO_4_^−^ can impair the proper function of thyroid gland. Singh [40] developed a fluorescence turn-off sensor, PDI-28 (Figure 9b), which undergoes fluorescence quenching with only ClO_4_^−^ ions (forming 1:1 PDI-28-ClO_4_^−^ complex) in HEPES buffer (10 % DMSO, v/v, pH = 7.4). A LDL of 60 nM was determined under an optimal testing condition. The same sensor can also be used for solid phase detection of ClO_4_^−^ by doping the PDI-28 on TLC plate. For testing the broad practical applications of PDI-28 sensor, the detection of ClO_4_^−^ was also explored for drinking water and firework samples, as well living cell imaging.

#### 2.2.3. Cyanide Ion (CN^−^)

Cyanide anion is considered as the most toxic, lethal ion to living organism. Mukhopadhyay [41] developed a unique multi-modal sensor molecule PDI-29 (Figure 9c) for detection of CN^−^ in THF or THF/H_2_O (97/3, v/v) mixture solvents. Trifluoromethylbenzene groups are attached at the imide-sites of PDI in order to lower the energy level of lowest unoccupied molecular orbital (LUMO), thus enhancing the electron-affinity of PDI core. Upon reaction with CN^−^, an air-stable anionic radical of PDI, PDI-29^•^^−^, can be generated through a single-electron transfer process (Figure 9d). Combination of the spin, charge and the singly occupied molecular orbital (SOMO)-LUMO-based electronic transition, the anionic radical thus formed can be used to produce multi-modal sensing signal for detection of CN^−^ with high selectivity. As shown in Figure 9d, the multi-modal sensor can be realized through reversible color change (colorimetric sensing). The fluorescence quenching accompanied with the color change led to high sensitivity in detection of CN^−^ ion down to the level of 0.2 µM (5 ppb) to 3.5 µM (87 ppb) in THF media.

#### 2.2.4. Phosphate (PO_4_^−^)

Fundamental phosphate compounds like adenosine triphosphate (ATP) and one of its hydrolyzed substances, pyrophosphate (PPi), are related to some severe environmental and health issues. Efficient detection of PO_4_^−^ by PDI-based optical sensors is often based on fluorescence enhancement, and the binding of PO_4_^−^ is usually through coordination with a metal ion site pre-attached at the PDI. Attachment of metal ions at PDIs can be achieved by substituting the metal complex ligands like carboxylate or picolylamine at the imide-positions [42,43,44,45]. These complexligands are not fluorescent by themselves (not causing background interference), and modification at the imide-positions does change the fluorescence property of PDI. 

**Figure 10:**
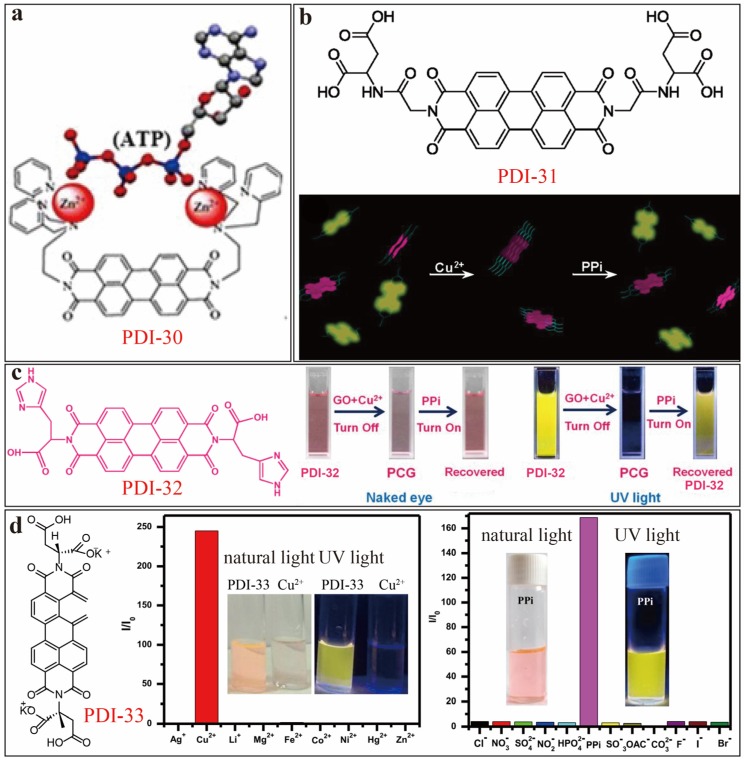
(**a**) Schematic illustration of sensing mechanism of PDI-30-Zn^2+^ towards ATP. Reproduced with permission from [42], copyright 2012 Elsevier B. V. (**b**) Molecular structure and schematic assembly disassembly process of PDI-31 upon addition of Cu^2+^ and PPi subsequently. Reproduced with permission from [43], copyright 2012 American Chemical Society. (**c**) Molecular structure of PDI-32, and photographs of color and fluorescence emission changes of PDI-32 (10 µM) solutions after adding Cu^2+^ (20 µM) with GO (10 µg/mL), and PPi (10 µM) in sequence, under natural light or UV light (λ_ex_ = 254 nm). Reproduced with permission from [44], copyright 2017 Elsevier B. V. (**d**) Molecular structure of PDI-33, and photographs of its solutions (10 µM in aqueous HEPES buffer solution, pH = 7.4) before and after adding Cu^2+^ (100 µM) and PPi (100 µM) under natural light and UV light (λ_ex_ = 365 nm), as well as their selective fluorescence enhancement response under λ_ex_ = 440 nm. Adapted and reproduced with permission from [45], copyright 2019 American Chemical Society.

Yan [42] constructed an ATP sensor PDI-30 (Figure 10a), which is modified with a Zn^2+^–dipicolylethylenediamine (Zn^2+^–DPEN) moiety at the two imide-positions. The sensing mechanism is primarily based on complexation between ATP and the two Zn^2+^ centers. The fluorescence of PDI-30 is intrinsically weak in aqueous solution, but it gets significantly enhanced upon addition of ATP. Other phosphate anions were also tested under the same conditions, but none of them showed fluorescence enhancement as comparable as ATP, implying high detection selectivity. Similarly, Cu^2+^ ion can also be used to mediate the sensing of phosphate, i.e., competitive binding between PO_4_^−^ and Cu^2+^ leads to disruption of the aggregate of PDI-Cu^2+^ complex, which in turn turns on the fluorescence. One of such examples was developed by Li [43] based on PDI-31 (Figure 10b). PDI-31/Cu^2+^ complex (1:2) tends to form aggregate in pure aqueous solution, leading to the fluorescence quenching. Addition of PPi into the solution caused disassembly of the aggregate due to the competitive binding of PPi with Cu^2+^, which in turn resulted in recovery of PDI fluorescence. The fluorescence turn-on sensor was proven sensitive and selective toward PPi (against other common anions), with a *LDL* of 0.2 μM. Later, Iyer [44] reported on a unique three-component nanocomposite sensor system, namely PCG as shown in Figure 10c, which represents a self-assembly of histidine-functionalized PDI-32, graphene oxide (GO) and Cu^2+^ ions. PCG demonstrated efficient detection of PPi via the same fluorescence turn-on mechanism as described above, and is suite for detecting PPi in water under physiological conditions, as well as in vitro monitoring in living cells. A *LDL* of 60 nM was determined, and the high sensitivity is mainly due to the strong binding affinity between the copper complex of PDI-32 and PPi. Moreover, the PCG sensor can be expended from solution to solid phase detection, for example by fabricating the PCG composite with PVA hydrogel films and on thin-layer chromatography (TLC) plates, which represent more practical utility for the detection of PPi in a label free manner. Very recently, Sukul [45] reported a similar sensor platform based on PDI-33-Cu^2+^ aggregates (Figure 10d), which exhibited significant sequential fluorescence turn-off and turn-on responses toward Cu^2+^ and PPi, respectively. PDI-33 was found to be selective toward Cu^2+^ and PPi over other phosphates including adenosine monophosphate, adenosine diphosphate, and adenosine triphosphate (ATP). Addition of Cu^2+^ into the solution of PDI-33 led to significant fluorescence quenching of PDI, whereas no quenching was observed for other metal ions. The non-fluorescent PDI–Cu^2+^ ensemble thus formed can be turned on its fluorescence upon binding to PPi. The detection of PPi, as clearly unveiled by the change in color of the solution, can simply be monitored by naked eyes. A *LDL* of 0.11 μM was estimated for PPi.

### 2.3. Organic Pollutant Sensing

PDI-based optical sensors have proven efficient for detection of organic compounds of biology interest, e.g., ascorbic acid [46] and thyroid hormones [47], in aqueous media. With the similar sensing mechanism and molecular design strategy, PDI-based sensors have also been adapted into detection of toxic organic compounds, such as hydrazine, other amines and nitroaromatics [48,49,50,51,52,53,54]. These compounds pose toxicity and pollution problems to water environment and human health. It remains crucial to develop portable, low-cost chemosensors that are suited for quick, on-site detection of organic compounds with both high sensitivity and selectivity (essential for minimizing false negatives and false positives).

#### 2.3.1. Hydrazine

**Figure 11:**
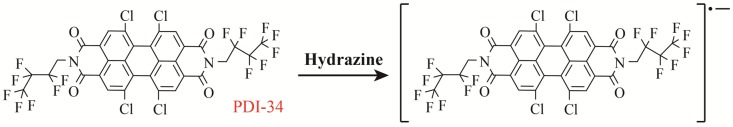
Molecular structures of neutral and one-electron reduced PDI-34.

Hydrazine (H_2_N-NH_2_) is a common industry reagent, and considered highly toxic with potential damage to human organs including central nervous system, respiratory system, liver and kidney. It is also a suspected human carcinogen. Detection of hydrazine has been researched all the time, but still remains a big challenge, mainly because of the interference from other organic amines that possess similar binding and redox property as hydrazine. Taking advantage of the high reactivity of hydrazine, many PDI-based sensors are designed to be a fluorescence turn-on sensor triggered by reaction with hydrazine. This is in contrast to the above-mentioned fluorescence sensors based on either PET of assembly-disassembly process, which both depend on non-covalent interaction, rather than permanent chemical reaction. Additionally, the strong reduction power of hydrazine can also be used to design some unique sensor, for example, single-electron reduction of PDI produces anionic radical in distinct green color, which can be taken as a colorimetric sensing signal [48]. Stronger reducing reagents like LiAlH_4_ can even convert the PDI to the tetracarboxyl-eliminated derivatives [49]. Taking on the hydrazine reduction reaction, Zhang [48] developed multi-modal sensor based on PDI-34 (Figure 11) for trace detection of hydrazine. Bay substitution with four chlorines of PDI-34 further increases the electron affinity (oxidation power) of PDI core, which is conductive for sensing hydrazine. A dual-mode sensing can be obtained in a DMF solution involving both color change from yellow to green (characteristic of anionic radical formation) and fluorescence quenching. However, the decrease in fluorescence intensity was only 6.8% towards 0.025 equiv. hydrazine, meaning low sensitivity for the sensor regarding practical application.

#### 2.3.2. Amines

Organic amines are main industrial chemicals, and used widely in medicines and drugs, as well as some explosives. The extensive use of amines makes them to become common pollution to environment ranging from water to air, and to many other scenarios. Although great deal of effort has been put in the development of chemosensors for amines, detecting these chemicals, particularly at trace level, still remains challenging. The high diversity of amines regarding both structure and chemical property makes the sensor development even harder especially when certain detection specificity is required. Organic amines can be primary, secondary, tertiary or aromatic amines considering the substitution at the nitrogen, and the similarity in binding and reduction reaction (electron transfer) is the main reason behind the difficulty for sensing discrimination. Though it is impossible to detect or identify an individual amine with chemosensor, certain type of class of amines, e.g., aliphatic amines, can be distinguished from other analogues taking leverage on the relatively distinct structure and chemical property of aliphatic chains. Among all the amines studied thus far, aniline and its derivatives are the mostly researched mainly because that aniline is a major industry chemical and imposes serious environmental and healthy impact. 

**Figure 12:**
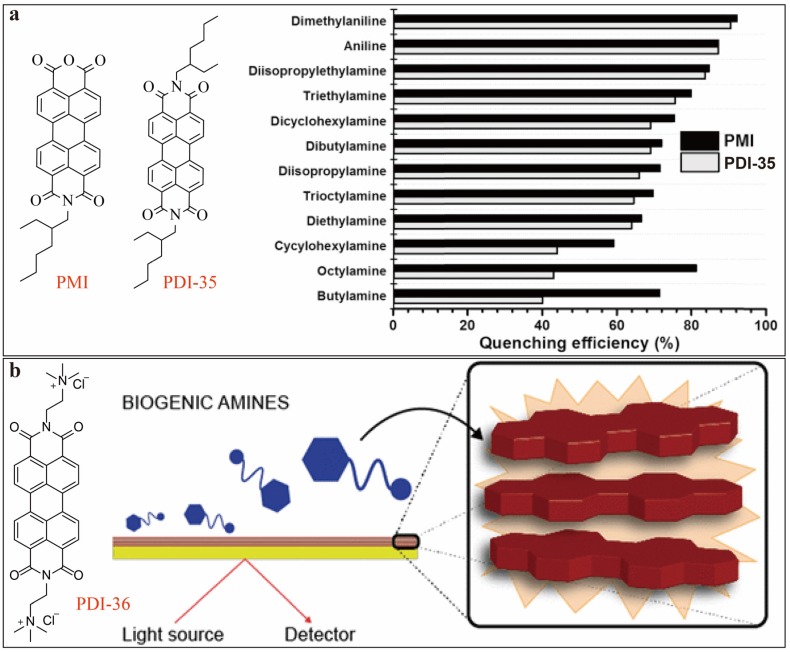
(**a**) Molecular structures of PDI-35 and PMI, and their fluorescence quenching efficiency towards various amines in THF solutions. Reproduced from [51], copyright 2016 Elsevier B. V. (**b**) Molecular structure of PDI-36, and schematic illustration of its sensing mechanism to biogenic amines. Reproduced from [52], copyright 2019 American Chemical Society.

Along with other popular chemosensors, molecules of PDI and perylene monoimide (PMI, shown in Figure 12) have been widely researched for detection of amines. Typically, intermolecular electron transfer from an amine to the highest occupied molecular orbital (HOMO) of singlet excited state of PDIs leads to fluorescence quenching of PDIs either in solutions or solid phase. [50] One such case of study as shown in Figure 12a was reported by Valiyaveettil [51] on PDI-35 and an alkyl substituted PMI, which showed efficient fluorescence quenching upon addition of various amines (including primary, secondary and tertiary amines). Overall, PMI exhibited better sensitivity than PDI-35, due to the strong binding affinity of anhydride group toward amines, but in general, PDI-35 showed higher selectivity for bulky tertiary/aromatic amines owing to their hydrophobic properties and favorable energy difference with HOMO level (−5.99 eV) of PDI-35 over linear primary amines, for example, dimethylaniline (−5.02 eV), aniline (−5.39 eV), diisopropylamine (−5.74 ev) and butylamine (−6.21 eV). This provide PDI-35 and PMI as promising sensors for detecting bioamines such as phenylethylamine, putriscine, spermine, spermidine and diethylenetriamine (DETA) in THF solutions, with potential aim to monitor food freshness and health status. The observed fluorescence quenching can be recovered by adding acid, which in turn protonates the amines, thus disabling their electron donating (fluorescence quenching) capability. 

In attempting to discriminate aromatic amines from other types of amines, Prato and Giancane [52] developed very recently a π–π stacked tubular aggregate self-assembled from PDI-36 (Figure 12b). 

The electronic interaction between PDI-36 aggregates and biogenic amines leads to modulation of the fluorescence of PDI, which in turn can be used as a sensing mode for detecting amines. Depending on different types of amines, the sensing mechanism could be different, enabling discrimination of different classes of amines. For example, fluorescence enhancement was observed upon interacting with phenylethylamine through amine-intercalated partial disaggregation of the π−π aggregates. With the same sensing mechanism, lower sensitivity was observed for putrescine because it has no aromatic ring (thus causing less significant disaggregation of PDI aggregates). In contrast, fluorescence quenching was observed when interacting with histamine, tryptamine, and tyramine, mainly due to the increased electron donating power of these amines. A *LDL* of as low as 0.1 nM was obtained for the detection of biogenic amines under aggregation/disaggregation sensing mechanism in aqueous solution.

#### 2.3.3. Nitroaromatics

**Figure 13:**
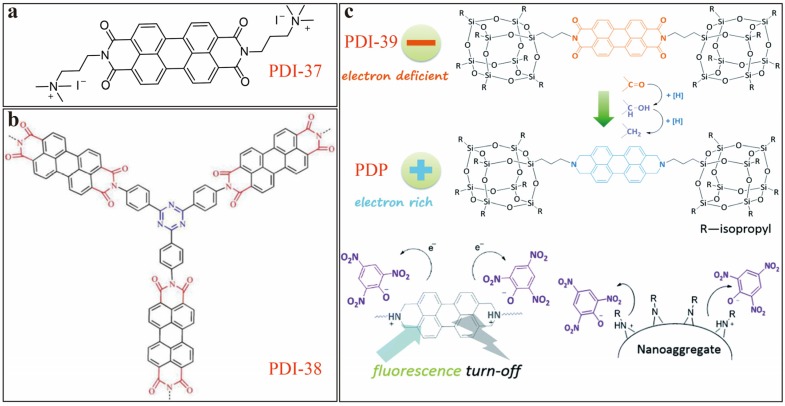
(**a**) Molecular structure of PDI-37. (**b**) Molecular structure of PDI-38. (**c**) Illustration of molecular structures of PDI-39 and PDP, and PA-induced fluorescence quenching mechanisms of PDP in solution and solid state. Adapted and reproduced with permission from [49], copyright 2015 Royal Society of Chemistry.

Nitroaromatic chemicals such as nitroaniline (NA), nitrophenol (NP), picric acid (PA), etc. bring severe contamination in water and soil, and explosive harm in atmosphere. Madhu (2016) [53] reported on PDI-37 sensor (Figure 13a), which is substituted with two ethelenetrimethyl ammoniumiodide groups. PDI-37 is soluble in water, and acts as a fluorescence quenching sensor for detection of 4-nitroaniline (4-NA) and PA with decent selectivity over other interference nitroaromatics as tested in DMF and neutral aqueous solutions. Compared to the normal PDIs, the salt of PDI-37 exhibits extremely stable fluorescent emission in DMF and water (within wide pH range from 1.0 to 10.0). A *LDL* of 1 μM was determined, which is low enough for practical environmental monitoring. Similar fluorescent quenching detection of nitrophenols has also been investigated in organic solvents using a rigid PDI trimer structure, namely PDI-38 (Figure 13b), as reported by Geng [54]. PDI-38 possesses strong molecular fluorescence emission, which can be significantly quenched upon interacting with *o*-nitrophenol (*o*-NP) in chloroform (CHCl_3_), which gives a *LDL* of 0.017 nM. The mechanism of fluorescence quenching is due to aggregation of PDIs caused by the strong complexation of *o*-NP with PDI-38 via hydrogen bonding. To detect electron-deficient nitroaromatic compound like PA, Zhang [49] reported a fluorescence quenching sensor based on POSS-functionalized PDI, namely PDI-39 (Figure 13c). 

First, a PDI substituted with two POSS was synthesized, followed by reduction of the four carbonyl groups of PDI backbone to form PDP. The two electron-rich tertiary amine centers of PDP enable strong response to electron-deficient PA, which in turn forms hydrogen bonds with the hydroxyl of PA in aprotic solvents. PDP exhibits colorimetric and ratiometric fluorescence quenching to PA in THF, which results in selective and sensitive sensing. The nanoaggregates of PDP fabricated by interfacial assembly also demonstrate fluorescence quenching upon drop-casting PA solution in THF, for which a *LDL* was estimated as 10^−15^ M, which is extremely low compared to many other chemosensors. The structure design presented in this study provides new guideline for developing next generation of sensors for PA. 

## 3. PDIs-Based Colorimetric Sensors for Environment Detection

Colorimetric sensing is of particular interest for environmental detection and monitoring because of its ease and simplicity of operation, low cost and expedient readout (even with naked eyes) without using additional instruments. PDI-based colorimetric sensors have been utilized for probing environmental factors such as humidity, solvent polarity, pH, and metal and non-metal ions, organic pollutants contained in environment [4,5,6,55]. Some PDI sensors were even used for producing biometric fingerprints [56]. 

**Figure 14:**
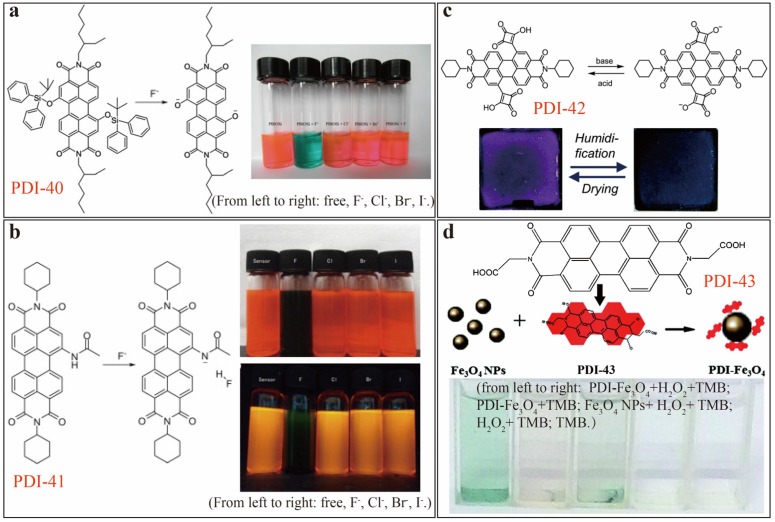
(**a**) Molecular structures of PDI-40 and its product in the presence of F^−^ anion showing different color change in THF solution containing 1.0 molar equiv. tetrabutylammonium salts. Reproduced with permission from [57], copyright 2012 Wiley. (**b**) Molecular structure of PDI-41 and corresponding F^−^ ion-mediated intermolecular proton transfer, with the photographs showing color and fluorescence change upon adding different halide anions. Reproduced with permission from [58], copyright 2012 Elsevier B. V. (**c**) Schematic illustration of the base/acid-triggered molecular structure interconversion between PDI-42 its deprotonated state, with photographs showing humidity-triggered reversible color change of PDI-42 in PEG matrix. Reproduced with permission from [59], copyright 2015 Royal Society of Chemistry. (**d**) Schematic structure of PDI-43-Fe_3_O_4_ nanocomposite, with photographs showing color change upon addition of hydrogen peroxide and other species. Reproduced with permission from [60], copyright 2017 the Centre National de la Recherche Scientifique (CNRS) and Royal Society of Chemistry.

As shown in Figure 14a, Wang [57] developed a PDI-40 colorimetric sensor for highly selective detection of F^−^ over other halides in THF solution. Rapid and distinct color change (from red to green) was observed for PDI-40 upon mixing with F^−^ ions, which is due to the specific cleavage of Si-O bond in PDI-40 by F^−^. Fluoride ion has strong reactivity toward the silicon site. Meanwhile, the same lab [58] also reported another colorimetric sensor system, based on PDI-41 (Figure 14b), which exhibits strong color change from red to dark green upon interacting with F^−^ion in dichloromethane (DCM) solution. Accompanying the color change, ratiometric fluorescence change was also observed that can be used as well as sensor signal modulation. Under the optimal testing condition, a *LDL* of 0.14 μM was obtained. The sensing mechanism of PDI-41 is based on an intermolecular proton transfer (IPT) process mediated by F^−^ ion as illustrated in Figure 14b. Compared to other halide ions, F^−^ tends to form much stronger hydrogen bond with the amide proton, thus enabling detection selectivity.

Maeda and Würthner [59] reported a colorimetric pH and humidity sensor based on PDI-42 (Figure 14c), which bears halochromic and hydrochromic squaric acid groups attached at the bay area. pH induced protonation/deprotonation of the hydroxyl groups of PDI causes strong color change as envisioned in both THF solution and polyethylene glycol (PEG) matrix (Figure 14c). The colorimetric sensing thus observed is due to the electronic change of the cyclobutene skeleton, which in turn results in dramatic electronic change in PDI core through direct conjugation between the cyclobutene and PDI. This pH sensing mechanism is reminiscent of the commonly used pH indicators [32,33,34,35,36,37,38]. Other molecular design strategies (some uncommon) have also been brought into this field. For example, as depicted in Figure 14d, Shi and Liu [60] developed an enzyme mimetic nanocomposite composed of magnetic Fe_3_O_4_ nanoparticles and PDI-43 modified with carboxymethyl side groups. This nanocomposite possesses peroxidase-like activity for colorimetric sensing of H_2_O_2_ and glucose, which gave a *LDL* of 2 µM and of 1.12 µM, respectively. The efficient sensing is mainly due to the high binding affinity between nanocomposite and the peroxidase substrate 3,3’,5,5’-tetramethylbenzidine (TMB), in synergy with the catalytic oxidation reaction involving H_2_O_2_. 

## 4. PDI-Based Multi-Modal Optical Sensors for Environment Detection

**Figure 15:**
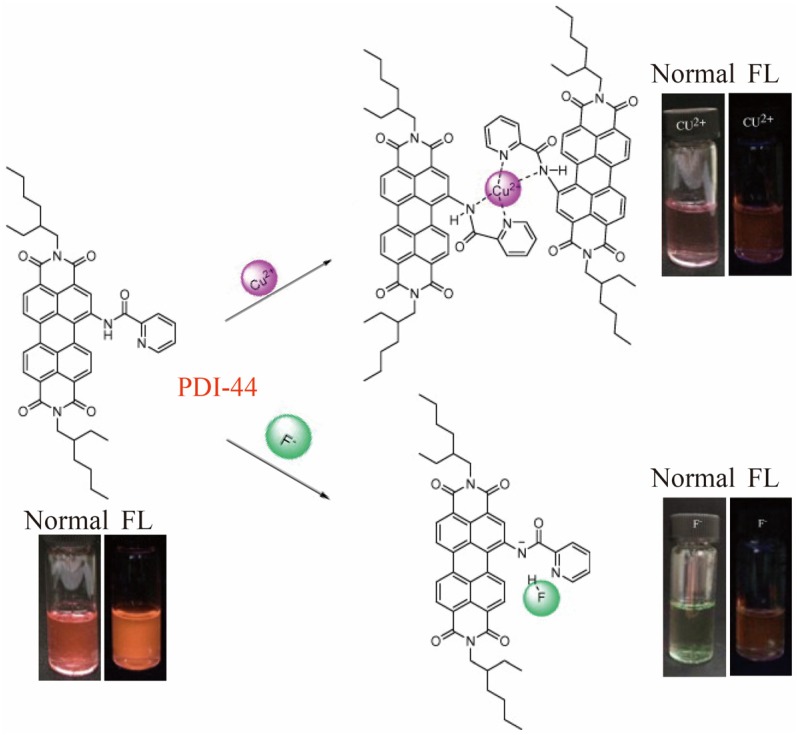
Schematic illustration of the sensing mechanisms of PDI-44 towards Cu^2+^ and F^−^ ions, also shown are the photographs of the corresponding solutions before and after adding ions, taken under natural light and UV illumination. Adapted and reproduced with permission from [61] copyright 2014 Elsevier B. V.

Single-mode chemosensors always face challenge in detection selectivity unless the binding receptor is designed with high specificity. The unique structural features of PDIs, especially the synthetic flexibility in side group modification at bay- and imide- position, provide PDI based sensors enormous options to be developed as multi-modal sensors with improved detection selectivity. As described above, fluorescence turn-on sensing (PDI-9) in combination with pH-adjustment of fluorescence enables discrimination between Zn^2+^ and Cd^2+^ [15], the two transition metal ions that are usually difficult to distinguish with many chemosensors. Combination of both fluorescence quenching and enhancement responses in signal processing leads to selective detection Hg^2+^ (PDI-17) [24]. Similarly, improved selectivity can be achieved by incorporating both colorimetric and fluorescence responses, which have been employed for detecting various cations and anions as described above [8,9,14,49,58]. Some innovative molecular design of PDIs could make one single sensor to be capable of detecting simultaneously different cations and anions. As presented in Figure 15, Wang (2014) [61] reported an interesting PDI-44-based chemosensor, which exhibits not only colorimetric response, but also dual-mode fluorescence modulation. Combining all these sensing responses, this sensor was successfully developed for selective and sensitive detection of both Cu^2+^ and F^−^ ions. Sensing of Cu^2+^ relies on the color change from rose red to purple, accompanied by fluorescence quenching as observed in THF/3-(N-morpholino)propanesulfonic acid (MOPS) buffer (4/1, v/v, pH = 7.2) medium (with *LDL* of 0.17 μM). The sensing mechanism is attributed to the formation of PDI-44-Cu^2+^-PDI-44 complex, which quenches the fluorescence of PDI. In comparison, sensing of F^−^ is realized with a different color change from rose red to light green, which is also accompanied by quenching of fluorescence emission as observed in THF solution (with *LDL* of 22 µM). But the sensing mechanism of F^−^ is based on the intermolecular proton transfer mediated by fluoride ions. [58].

## 5. Conclusion and Perspectives

In summary, as presented in Table 1, PDI-based fluorescence and colorimetric sensors, as well as the related sensor principles and signal modulation modes have been proven successful for detecting poisonous ions and organic compounds in liquid-phase environment with high selectivity and sensitivity (with *LDL* down to nM or sub-ppb) within wide linear detection range (*LDR*). These poisonous species impose serious threats to the human health and other biological systems associated water (and the related ground water or soil). However, the current chemosensors, despite great progress made (with some even commercialized), there are still a lot of rooms at the bottom for molecular design to further improve the sensing performance, especially the sensing selectivity. The challenge of addressing selectivity is mainly referred to the discrimination between the metal ions (e.g., those from the same periodical group) or analogous compounds (e.g., amines), which possess the similar binding and chemical interaction property. Though molecular design of specific binding group may help enable certain level of detection selectivity, creating a sensor system or platform that incorporates multiple sensing modes (e.g., fluorescence quenching, enhancement, ratiometric fluorescence modulation at two different wavelengths, color change, etc.) would provide great potential for future development of chemosensors. Surely the development of multi-modal sensor system will also have to be relied on the signal integration and optimization, which in turn will demand interdisciplinary approach capitalizing on the effort from electrical and mechanical engineering, data analytics and algorithm, and among others.

An alternative way that holds great feasibility in improving the detection selectivity is to construct a sensor array by incorporating multiple sensor units in a chip or microfluidic system. By incorporating all the sensing signals from each of the sensors (e.g., signal rise or decay, response or recovery rate, relative response magnitude under the same condition, etc.), an array will enable differential sensing that can be used to achieve high degree of discrimination between even chemical analogues (for similar metal ions or same type of organic compounds) by using appropriate algorithm methods, like machine learning based decision tree. The array-based differential sensing is highly reminiscent of the tasting system of mammals, for which the large number of tasting buds on tongue function like a sensor array. PDIs are ideal candidates for development as chemosensor array taking advantage of the structure flexibility and ease in substitution modification at both the sides and bay area as described above in this review. Wide range option of different binding and electronic structures of PDIs would allow enormous opportunity for enabling or enhancing the detection selectivity.

In addition to the great extensibility for development as diverse modes of chemosensors targeting broad range of environment analytes, PDI-based materials also possess other unique features, such as remarkably strong thermal/chemical/photochemical stability, environment benignity, good biocompatibility, and low cytotoxicity [3,37,62,63,64]. Indeed, due to these superior features (compared to most of other chemosensors), PDI-based fluorescence and colorimetric sensors, as well as the related molecular probes, have already been extensively employed in physiological and food detections [62], cell imaging and related biological probing [22,30,33,35,37,40], and biomedical imaging and photodynamic therapy [63,64]. Moreover, the great success of PDI-based sensors in liquid-phase detection can be extended onto development of solid-phase sensors, e.g., thin films, which will further mitigate the risk of toxicity impact to the environment. Solid phase sensors will not only prevent dissolution of PDIs into water system, but more importantly will enhance the technical feasibility of recycling the materials and thus the sustainability in practical use.

## Figures and Tables

**Table 1:** Sensor performance of PDI-based optical chemosensors toward diverse analytes under optimized testing conditions and related sensing principles ^1^.

PDIs	Basic Sensing Principles	Sensor Signals	Conc. of PDIs (µM)	Testing Solutions (v/v)	Targeted Analytes	*LDR* (µM)	*LDL* (µM)	Ref.
PDI-1-Au NPs	metal coordination	FL off-on	1.0	CHCl_3_/CAN (9/1)	Cu^2+^	0–100	1.0	[7]
PDI-2	metal coordination	color change, FL on-off	10	H_2_O/THF (7/3)	Cu^2+^	0–100	-	[8]
PDI-3	metal coordination	color change	10	CHCl_3_	Cu^2+^	0–100	0.50	[9]
		FL on-off				0–100	1.0	
PDI-3-Cu^2+^	metal coordination	color change	10	CHCl_3_	CN^−^	0–100	10	
		FL off-on				0–100	8.0	
PDI-4	host–guest-driven aggregation-deaggregation	FL on-off	3.5	aqueous CTAB microemulsion	Fe^3+^, Cu^2+^	0–21, 0–7.0	-	[10]
PDI-5	metal coordination	color change, FL off-on	0.050	ACN	Cu^2+^	0–10	0.020	[11]
PDI-6	metal-crown ether- induced aggregation	FL on-off	11	ACN	Ba^2+^	0–74.8	-	[12]
PDI-7	metal coordination	FL off-on	1.0	ACN	Al^3+^	0–5.0	0.33	[13]
PDI-8	Lewis acidic protonation	color change, FL on-off	mM	DMF	Fe^3+^, Al^3+^	µM–mM	µM	[14]
PDI-9	metal coordination	FL off-on	1.0	ACN/HEPES buffer (1/1)	Zn^2+^ (pH 6.0–7.0)	0.1–4.0	0.032	[15]
					Cd^2+^ (pH 9.0)	0.1–5.0	0.048	
PDI-10	metal coordination	FL off-on	10	Tris-HCl buffer, pH 7.3	Cd^2+^	0–600	0.52	[16]
PDI-11	metal coordination	FL on-off	0.10	DMF/H_2_O (7/3)	Hg^2+^	0–1.0	0.0050	[17]
PDI-Hg^2+^	thymine-Hg^2+^ complexation	FL off-on	0.33	DMF/H_2_O (9/1)	cysteine	0.05–0.3	0.0096	[18]
PDI-12	J-aggregation	color change, FL on-off	1.0	THF/H_2_O (2/1)	Hg^2+^	0.1–2.0	0.037	[19]
	deaggregation	color change, FL off-on			cysteine		0.091	
PDI-13	metal coordination	FL on-off	50	DMSO/HEPES buffer (1/19), pH = 7.4	Hg^2+^	0–250	0.0060	[20]
PDI-13-Hg^2+^	thymine-Hg^2+^-induced deaggregation	FL off-on			biothiols (Cys, Hcy, GSH)	0–400	0.0015, 0.0083, 0.0011	
PDI-14	thymine-Hg^2+^-induced deaggregation	FL off-on	30	pure water	Hg^2+^	0–30	0.10	[21]
PDI-15	metal coordination	FL off-on	2.0	DMF/H_2_O (19/1), pH = 5.5–7.5	Hg^2+^	0–200	0.56	[22]
PDI-16	metal coordination	FL off-on	20	DMF/H_2_O (19/1)	Hg^2+^	0–1000	2.2	[23]
PDI-17	metal coordination	FL off-on at 365 nm FL on-off at 557 nm	5.0	DMSO/Na_2_HPO_4_ buffer (1/1), pH 7.0	Hg^2+^	1.0–20	0.010	[24]
PDI-18	metal-tetraester coordination	FL off-on (FE 184)	2.0	DMF/H_2_O (1/1), pH 2–10	Fe^3+^	1.0	-	[25]
PDI-19	metal-polyamidoamine coordination	FL off-on (FE 6.4)						
PDI-20	metal-EDTA coordination	FL off-on (FE 1.83)	5.0	DMSO/H_2_O (1/1)	Fe^3+^	50	-	[26]
		FL off-on (FE 1.18)			Al^3+^			
PDI-21	metal-DTA coordination	FL off-on (FE ~7)	2.0	ACN/H_2_O (1/1)	Fe^3+^	18	-	[27]
PDI-22	metal-DPA coordination	FL off-on (FE 49)	6.0	DMF	Ni^2+^	84	-	[28]
PDI-23	metal-EDPA coordination	FL off-on (FE 138)	5.0		Fe^3+^	20	-	
PDI-24	metal coordination	FL off-on	5.0	DMF/H_2_O (7/1)pH 2 (HCl)–10 (NaOH)	Pd^2+^	0-15 ppm	0.0073	[29]
PDI-25	depropargylation reaction	NIR color change, FL on-off at λ_em_ 630 nm	10	DMSO/HEPES buffer (1/9), pH 7.3	Pd^0^	μM	0.0066	[30]
		NIR color change, FL on-off at λ_em_ 564 nm		THF/HEPES buffer (1/1), pH 7.3			0.021	
PDI-26	Tsuji–Trost allylic oxidation and decarboxylation	NIR color change	1.0	DMSO/HEPES buffer (1/1), pH = 7.2	Pd^0^	0–40	0.039	[31]
		FL on-off				0–90	0.045	
PDI-27	F^−^inducded hydrolyzation of POSS nanocages	FL on-off	30	pure water	F^−^	0–1000	10	[39]
PDI-28	ion complexation	FL on-off	10	DMSO/HEPES buffer (1/9), pH = 7.4	ClO_4_^−^	0–70	0.060	[40]
PDI-29	SOMO-LUMO-based eT	color change, FL on-off	0.15	THF	CN^−^	0.2–3.5	0.20	[41]
PDI-30	Zn^2+^-PO_4_^−^ complexation	FL off-on	10	HEPES buffer. pH 7.4	ATP	0–20	-	[42]
PDI-31	Cu^2+^-PO_4_^−^ complexation	FL off-on	5.0	HEPES buffer, pH 7.4	PPi	0.1–30	0.20	[43]
PCG	PDI-32+GO+Cu^2+^	FL off-on	0.33	HEPES buffer, pH 7.4	PPi	0–0.33	0.060	[44]
PDI-33	Cu^2+^-PO_4_^−^complexation	color change, FL off-on	10	HEPES buffer, pH = 7.4	PPi	40–100	0.11	[45]
PDI-34	reduction reaction	color change	10	DMF	hydrazine	0.32–2.90 nmol	0.87 nmol	[48]
		FL off-on				0.65–3.57 nmol		
PMI	intermolecular electron transfer	FL on-off	0.10	THF	amines	100–10^6^	-	[51]
PDI-35								
PDI-36	amine-intercalated disaggregation	FL off-on	40	deionized water, pH ≈ 8	bioamines	10^−4^–100	10^−5^	[52]
	intermolecular electron transfer	FL on-off			bioamines			
PDI-37	H-bonding-induced aggregation	FL on-off	μM	DMF; aqueous solutions, pH 1.0–10.0	4-NA, PA	0.1–1.0	up to 1.0	[53]
PDI-38	H-bonding-induced aggregation	FL on-off	1.7 × 10^3^	CHCl_3_	*o*-NP	0–750	1.7 × 10^−5^	[54]
PDP (reduced PDI-39)	H-bonding-induced aggregation	color change, FL on-off	1.5	THF	PA	0–18	-	[49]
PDI-40	Si-O bond cleavage	color change	50	THF	F^−^	0–10	-	[57]
PDI-41	intermolecular proton transfer	color change, FL on-off	10	DCM	F^−^	0–120	0.14	[58]
PDI-42	protonation/deprotonation	color change	-	THF/H_2_O	pH; humidity	-	-	[59]
PDI-43-Fe_3_O_4_ NPs	peroxidase substrate binding	color change	-	aqueous solutions	H_2_O_2_	7.0–100	2.0	[60]
					glucose	3.0–100	1.1	
PDI-44	metal coordination	rose red to purple, FL on-off	10	THF/MOPS buffer (4/1), pH = 7.2	Cu^2+^	0–10	0.17	[61]
	intermolecular proton transfer	rose red to light green, FL on-off	10	THF	F^−^	0–15	Up to 22	

^1^ The data of *LDL*, highly dependent on the concentration of PDI sensor used, were determined under the optimized testing conditions. The data of *LDR*, in some cases, were just concentration range actually studied and reported in the referred literatures.
